# Prevalence of Dementia and Its Associated Risk Factors Among Geriatric Patients Visiting Primary Healthcare Centers in Riyadh, Saudi Arabia: A Cross-Sectional Study

**DOI:** 10.7759/cureus.24394

**Published:** 2022-04-22

**Authors:** Faisal M Alsebayel, Abdulaziz M Alangari, Fakhriya H Almubarak, Rana Alhamwy

**Affiliations:** 1 Family Medicine, King Fahad Medical City, Riyadh, SAU

**Keywords:** ad8, geriatrics, alzheimer’s dementia, screening tool, primary health care centers, depression, cognitive impairment and dementia, geriatric patient

## Abstract

Introduction: Dementia is a major cause of morbidity and dependence. The number of elderly individuals living with dementia worldwide is expected to rise up to 131 million by 2050. The Middle East and North Africa (MENA) is estimated to be one of the highest prevalence regions. However, there are limited numbers of studies in the region, especially on the primary care level. This study aims to determine the prevalence of dementia and identify the most important modifiable risk factors in our sample.

Methods: A cross-sectional study design was used utilizing the non-probability convenience sampling technique. A questionnaire including demographic data, the Arabic version of the Patient Health Questionnaire (PHQ9) to screen for depression, and the Arabic version of the eight-item Alzheimer's Dementia (AD8) to screen for dementia, were administered to participants recruited from six primary healthcare centers in Riyadh city, Saudi Arabia.

Results: This study found the prevalence of dementia to be 16% and 11% assuming cutoff points of 3 and 4, respectively, using the Arabic version of AD8, with depression and dyslipidemia as important modifiable risk factors for dementia in our sample.

Conclusion: Primary healthcare professionals should be aware of the important modifiable risk factors such as dyslipidemia and depression in the population to be able to develop strategies for early detection and slowing down the progression of dementia. Further research to identify other modifiable risk factors in the population is recommended.

## Introduction

Dementia is a term used to describe multiple brain disorders that affect memory, thinking, and behavior causing significant morbidity and dependence in the older population. It is estimated that over 55 million elderly individuals are living with dementia worldwide, a number that is estimated to rise to 131.5 million by 2050 [1,2]. Mild cognitive impairment (MCI) is recognized as a stage between normal age-related cognitive decline and dementia with an estimated annual conversion rate of 20% [3]. Strategies to slow down the progression of dementia include modifying risk factors as well as applying cognitive training programs [4,5]. There is a limited number of studies evaluating the prevalence of dementia in the Middle East and North Africa (MENA), though it is estimated to have one of the highest regional estimates in the world [2]. In Saudi Arabia, there is little information on the prevalence of dementia. In one study, the prevalence was 38.6% and 6.4% for MCI and dementia, respectively [6]. This high prevalence, according to the author, was attributed to the level of education, years of formal study, and other risk factors such as hypertension, diabetes, and dyslipidemia [6]. The current study aims to build on the previous studies in regard to determining the prevalence of dementia and identifying the most important modifiable risk factors by targeting a large sample size from six different primary healthcare centers in Riyadh city, Saudi Arabia. We used the validated Arabic version of the eight-item Alzheimer's Dementia (AD8) screening questionnaire to distinguish dementia from normal cognitive decline [7]. It is an informant-based brief questionnaire that has been argued to be a more appropriate form of dementia screening in the primary healthcare setting compared to the Mini-Mental State Examination (MMSE) [8].

## Materials and methods

In this cross-sectional study, participants were recruited from six different primary healthcare centers located in Riyadh city, Saudi Arabia in the period between November 2021 and February 2022. Participants above the age of 60 years were included. Participants with suspected delirium or recent head trauma (less than three months) were excluded. This study was approved by the institutional review board at King Fahad Medical City in Riyadh, Saudi Arabia (IRB#20-124). Verbal consent was obtained from all study participants before administering the questionnaire that included demographic data, the Arabic version of the Patient Health Questionnaire (PHQ9), and the Arabic version of the AD8 using a cutoff point of ≥ 3 and ≥ 4, which proven to have excellent sensitivity and specificity [7]. The sample included 379 participants, collected utilizing a non-probability convenience sampling technique. Sample size calculation was based on the prevalence of cognitive impairment from a previous study (approximately 45%), which is based on a population of 165,776 with a confidence level of 95% and a 5% margin of error [6,9].

Statistical analysis

The collected data were analyzed using IBM SPSS Statistics for Windows, Version 25.0 (Released 2017; IBM Corp, Armonk, New York, United States). A descriptive analysis was carried out and continuous variables were expressed as means ± SD or median. Categorical variables were summarized as frequencies and percentages. Binary logistic regression analysis was used to identify factors associated with dementia. Factors were selected with a backward stepwise method. Unstandardized regression coefficients (β) and odds ratios (ORs) and their 95% confidence intervals (CIs) were used to quantify the associations between variables. Statistically significant differences were considered when P < 0.05.

## Results

The study included 379 participants, of which 49.9% were males and 50.1% were females with a mean age of 68.2 years. As shown in Table 1, most participants were married and living with their families (71% and 89%, respectively). Half of the sample did not attend formal schooling for more than six years, 26.4% of them were illiterate, and 24.3% attended formal schooling for less than six years. Furthermore, 26.4% attended formal schooling for 7-12 years. The remaining 22.8% attended formal schooling for more than 12 years. Half of the sample were unemployed and 43.3% were retired. Most did not need assistance with daily activity (96%).

**Table 1:** Demographic data of studied population (n=379)

Demographic data of studied population (n=379)	Number	Percentage
Gender		
Male	189	49.9
Female	190	50.1
Marital Status		
Single	5	1.3
Married	272	71.7
Widowed	82	21.6
Divorced	20	5.3
Education level		
No formal schooling	100	26.4
< 6 years of formal schooling	92	24.3
7-12 years of formal schooling	100	26.4
> 12 years of formal schooling	87	22.8
Living Status		
Alone	34	9
With spouse	4	1.1
With family	340	89.7
Care facility	1	0.3
Employment status		
Employed	25	6.6
Unemployed	190	50.1
Retired	164	43.3
Income range (national average approximately 12000 Saudi Riyal)		
Above average	167	44.1
Below average	212	55.9
Assistance with daily activity		
Yes	15	4
No	364	96

Looking at the prevalence of risk factors in our sample we found that 8.2% had memory complaints and 9.7% had a family history of dementia. Diabetes prevalence was 59.8%, hypertension 58.8%, and dyslipidemia 35.8%. Around 10.5% were smokers and 53% reported exercising regularly, with 27.4% exercising more than 150 minutes a week as shown in Table 2. Depression prevalence in our sample was measured at 19.2% using the PHQ9 cutoff points, starting at 5% with mild depression, 5.3% with moderate, 6.4% with moderately severe, and 2.5% with severe depression. Using AD8 with a cutoff point of ≥ 2, we found the prevalence of dementia to be 24.5%; when applying a cutoff point of ≥ 3, it drops to 16.6%, and at a cutoff point of ≥ 4, it drops to 11.3%.

**Table 2:** Risk factors of studied population (n=379)

Risk factors of studied population (n=379)	Number	Percentage
Chronic Disease		
Yes	356	93.9
No	23	6
Diabetes		
Yes	227	59.8
No	152	40.1
Hypertension		
Yes	223	58.8
No	156	41.1
Dyslipidemia		
Yes	136	35.8
No	243	64.1
Hypothyroidism		
Yes	44	11.6
No	335	88.4
Ischemic heart disease		
Yes	34	9
No	345	91
Stroke		
Yes	14	3.6
No	339	89.4
Family history of dementia		
Yes	37	9.7
No	342	90.2
Smoking		
Yes	40	10.5
No	339	89.4
Exercise		
Yes	203	53.6
No	176	46.4
Regularly (>150 min/wks.)	104	27.4
Memory complaint		
Yes	31	8.2
No	348	91.8

Using binary logistic regression analysis, we found depression, age greater than 71, below-average income, and dyslipidemia as statistically significant risk factors for dementia in our sample with depression being the highest modifiable risk factor followed by low income and dyslipidemia as shown in Table 3. Other risk factors including diabetes, ischemic heart disease, hypertension, stroke, hypothyroidism, smoking, level of education, and family history of dementia were included in the analysis but found to be insignificant in our sample.

**Table 3:** Risk factor regression analysis (n=379)

Risk factor regression analysis for Dementia	P value	OR
Depression	< 0.001	5.045
Age Group	< 0.001	4.411
Income	0.004	3.185
Dyslipidemia	0.025	2.277

## Discussion

Based on limited research, it was estimated that the prevalence of dementia in the MENA is one of the highest worldwide at around 8.67% [2]. Our findings show high prevalence of dementia between 11.3% and 16.6% assuming cutoff points of 4 and 3, respectively, using the AD8 questionnaire. Comparing our findings to published studies in Saudi Arabia, Alkhunizan et al. published a study in 2018 including 171 participants using the Montreal Cognitive Assessment test (MoCA), which showed a prevalence of 38.6% and 6.4% of mild cognitive impairment and dementia, respectively [6]. Another study published in 2020 by Abd-El Mohsen et al. included 130 participants. Using the MMSE to predict mild and severe cognitive impairment, they found mild cognitive impairment to be 31.6% and severe cognitive impairment to be 17% [10]. We attribute the lower prevalence in our study to the sample being collected in the primary healthcare setting and using a screening method that doesn’t depend on the level of education of the study participants. Despite using different methodology, our results are in agreement with a recent study by Alshammari et al. in 2020, which used the MMSE in the same setting (primary healthcare) with 1299 participants. They found the prevalence of MCI to be around 17% and that of severe cognitive impairment to be 3.8% [11].

Prevalence of the studied risk factors in our sample was found to be higher than that of the general population (Table 2). Compared to the latest published data on the burden of disease in the elderly by the Saudi General Authority for Statistics, the prevalence of diabetes was reported to be 28.7%, hypertension at 28.5%, and dyslipidemia around 8% [9]. On the other hand, prevalence of depression in our sample was 19.2% compared to multiple studies reporting depression in elderly population ranging from 42% to 63.7% [12,13]. This finding in our study is significant considering the importance of depression as a risk factor for dementia.

Limitations

In this study, several factors may have affected the result and caused some limitations. First, a cross-sectional study design does not ensure a definitive causality of risk factors. Furthermore, the use of the convenience sampling technique although relatively inexpensive and easily achievable may introduce some sampling bias. Second, several risk factors including diabetes and hypertension have been established as risk factors when uncontrolled. However, in this study, we could not report on the level of control of these risk factors due to limited documentation in the healthcare information system of the primary healthcare centers. Finally, although the AD8 has been proven to be a reliable tool for the screening of dementia, more methods of assessment are required to make a definitive diagnosis and develop a management plan.

## Conclusions

This study found the prevalence of dementia to be between 11% and 16% in the primary healthcare setting. Depression was recognized to be an important modifiable risk factor as well as dyslipidemia. Awareness of these modifiable risk factors among healthcare professionals may help in early detection and, therefore, in slowing down the progression of dementia by prompt therapeutic interventions and other methods of treatment. Further research on a larger national scale is recommended to identify other modifiable risk factors for dementia.
